# A Comparison between Multiple Regression Models and CUN-BAE Equation to Predict Body Fat in Adults

**DOI:** 10.1371/journal.pone.0122291

**Published:** 2015-03-30

**Authors:** Pilar Fuster-Parra, Miquel Bennasar-Veny, Pedro Tauler, Aina Yañez, Angel A. López-González, Antoni Aguiló

**Affiliations:** 1 Department of Mathematics and Computer Science, Universitat Illes Balears, Palma de Mallorca, Baleares, Spain; 2 Research Group on Evidence, Lifestyles & Health. Research Institute on Health Sciences (IUNICS). Universitat Illes Balears, Palma de Mallorca, Baleares, Spain; 3 Instituto de Investigación Sanitaria de Palma (IdISPa), University Hospital Son Espases, Palma de Mallorca, Spain; 4 Prevention of Occupational Risks in Health Services, GESMA, Balearic Islands Health Service, Hospital de Manacor, Manacor, Spain; Faculty of Biology, SPAIN

## Abstract

**Background:**

Because the accurate measure of body fat (BF) is difficult, several prediction equations have been proposed. The aim of this study was to compare different multiple regression models to predict BF, including the recently reported CUN-BAE equation.

**Methods:**

Multi regression models using body mass index (BMI) and body adiposity index (BAI) as predictors of BF will be compared. These models will be also compared with the CUN-BAE equation. For all the analysis a sample including all the participants and another one including only the overweight and obese subjects will be considered. The BF reference measure was made using Bioelectrical Impedance Analysis.

**Results:**

The simplest models including only BMI or BAI as independent variables showed that BAI is a better predictor of BF. However, adding the variable sex to both models made BMI a better predictor than the BAI. For both the whole group of participants and the group of overweight and obese participants, using simple models (BMI, age and sex as variables) allowed obtaining similar correlations with BF as when the more complex CUN-BAE was used (*ρ* = 0:87 vs. *ρ* = 0:86 for the whole sample and *ρ* = 0:88 vs. *ρ* = 0:89 for overweight and obese subjects, being the second value the one for CUN-BAE).

**Conclusions:**

There are simpler models than CUN-BAE equation that fits BF as well as CUN-BAE does. Therefore, it could be considered that CUN-BAE overfits. Using a simple linear regression model, the BAI, as the only variable, predicts BF better than BMI. However, when the sex variable is introduced, BMI becomes the indicator of choice to predict BF.

## Background

Obesity is a chronic, multifactorial and complex disease defined as an excess in body fat [1]. Excess adiposity is associated with increased risk of type 2 diabetes, cardiovascular disease, fatty liver and others, leading all of them to increased morbidity and mortality [1–4]. Body fat (BF) can be measured by several techniques such as skin-fold measurements bioelectrical impedance analysis (BIA) and dual-energy X-ray absorptiometry (DEXA). However, most of these techniques are not available in the clinical practice or they are not adequate when large populations are considered. Therefore, different surrogate measurements of adiposity have been proposed, including the well-known Body Mass Index (BMI) and the most recent Body Adiposity Index (BAI). However, both of them, although easy to calculate, show important inaccuracies [5–8]. In order to find an accurate and simple estimator of BF, several prediction equations have been proposed. These equations commonly include weight and height as essential variables, but they also include variables such as age and sex to increase the adjustment of the predicted values to the practical measurements [6, 9–11]. Recently a new equation, the CUN-BAE equation, which includes BMI as the main variable has been suggested [12]. However, this equation seems to be very complex because includes up to nine terms. In this sense, the general *Ockham’s razor* principle, also called the principle of parsimony, recommends to choose the simplest hypothesis consistent with the observations, i.e., to use models and procedures that contain all that is necessary for the modelling but anything more. On the other hand, overfitting is the use of models that violates parsimony [13]. Furthermore, the CUN-BAE equation was obtained considering data from a population mainly overweight and obese [12]. Therefore, the aim of this study was to compare different multiple regression models to predict body fat, including the previous reported CUN-BAE equation and other simpler equations. The accuracy of these models in the whole group of participants and in the subgroup of overweight and obese participants was also compared.

## Methods

### Study design

A cross-sectional study with Caucasian adult workers (aged, 18 – 65 years) was performed. All subjects were from Mallorca (Spain) and belong to different productive sectors (public administration, health department, post offices).

Participants in the study were systematically selected during their work health periodic assessments between January 2008 and December 2010. Every day the first and the last examined worker were invited to participate in the study. 3,223 workers were invited to participate in the study. However, 23 refused to participate, being the final number of participants 3,200 (99.3%), with 1,726 women and 1,474 men. The mean age of participants in the study was 39.2 years (SD 0.19). Participants were informed of the purpose of this study before they provided written informed consent to participate. Following the current legislation, members of the Health and Safety Committees were informed as well. The study protocol was in accordance with the Declaration of Helsinki and was approved by the Institutional Review Board of the Mallorca Health Management (GESMA). To achieve the aims of this study two datasets were considered among participants in the study. The first one (S1 Dataset) includes all the participants in the study (n = 3,200). The second one (dataset B) was obtained considering only the overweight and obese participants (*BMI* > 25 *kg*/*m* ^2^, n = 1,498, 518 women and 917 men) in S1 Dataset. Criteria used to define overweight and obesity were the ones of the World Health Organization(WHO) [14].

*Anthropometrics*. All anthropometric measurements were made in the morning, after an overnight fast, at the same time (9 a.m.), and according to the recommendations of the International Standards for Anthropometric Assessment (ISAK) [15]. Furthermore, all measurements were performed by well trained technicians or researchers to minimize coefficients of variation. Each measurement was made three times and the average value was calculated. Weight and height were determined according to recommended techniques mentioned above. Body weight was measured to the nearest 1 kg using an electronic scale (Seca 700 scale, Seca gmbh, Hamburg). Height was measured to the nearest 1 cm using a stadiometer (Seca 220 (CM) Telescopic Height Rod for Column Scales, Seca gmbh, Hamburg). BMI was calculated as weight (kg) divided by height (m) squared (*kg*/*m* ^2^). Abdominal waist and hip circumferences were measured using a flexible steel tape (Lufkin Executive Thinline W 606). The plane of the tape was perpendicular to the long axis of the body and parallel to the floor. Waist circumference was measured half-way between the lower costal border and the iliac crest. The measurement was made at the end of a normal expiration while the subject stood upright, with feet together and arms hanging freely at the sides. Hip circumference was measured over nonrestrictive underwear or light-weight shorts at the level of the maximum extension of the buttocks posteriorly in a horizontal plane, without compressing the skin. The body adiposity index (BAI) was calculated using the equation suggested by Bergman and colleagues, *BAI* = (*hip* *circumference*)/((*height*)^1.5^) − 18). Percentage of body fat mass was obtained by Tetrapolar Bioelectrical Impedance Analysis (BIA) system (BF-350, Tanita Corp, Tokyo, Japan). BIA measurements were carried out at 50 kHz with a 0.8 mA since wave constant current under standard conditions. Whole-body composition was estimated using equations provided by the BIA manufacturer for all participants [15]. The reliability and validity of this system has been proved in Caucasian populations. BIA measurement using this methodology has been described in detail previously [16]. Subjects stood on the metal contacts in barefoot, and body fat mass was determined. This measurement was repeated twice, and the average value was obtained.

### Exploratory Analysis

Exploratory analysis was performed by examining tables and plots of the observed data. Exploratory analysis was used to (1) identify missing values, (2) verify the quality of the data, and (3) determine the terms used in the two regression models relating: i) body fat to BAI, and ii) body fat to BMI.

### Statistical Modeling

The general and anthropometric characteristics of the sample are presented as mean (SD). Body fat variable was normal distributed. BMI, BAI, and Age were logarithmically transformed, because of their non-normal distribution. Correlations between two variables were computed by Pearson (*ρ*) correlation coefficient. To relate body fat to BMI (models 1a, 2a, 3a, 4a, and 4’a using S1 Dataset, and model 1c, 2c, 3c, 4c, and 4’c using dataset B) and to BAI (models 1b, 2b, and 3b using S1 Dataset, and models 1d, 2d, and 3d using dataset B) we performed several standard multivariate linear regression models [17]. Model selection was performed on the basis of our exploratory analysis and prior knowledge of the relationship between gender and body fat. Coefficients were estimated with ordinary least squares and standard errors were calculated using standard asymptotic approximations [18].

### Validation Analysis

Validation of the obtained equations was performed using a new dataset (S2 Dataset) of 2,153 participants, with 753 women and 1,400 men. The mean age of participants in the sample used to validate results was 45.5 years (SD 8.0). Validation of equations was completed against BF values obtained experimentally using BIA as indicated above.

### Comparison with CUN-BAE equation

The comparison between the obtained equations and the CUN-BAE was made using the BF values from BIA. CUN-BAE equation applied was as follows [19]: *BF* = −44.988+(0.503×*Age*)+(10.689×*Sex*)+(3.172×*BMI*)−(0.026×*BMI* ^2^)+(0.181×*BMI*×*Sex*)−(0.02×*BMI*×*Age*)−(0.005×*BMI* ^2^×*Sex*)+(0.00021×*BMI* ^2^×*Age*) where male = 0 and female = 1 for Sex variable.

## Results

General and anthropometric characteristics of the sample are shown in Table 1.

**Table 1:** General and anthropometric characteristics of participants in the study.

	**All**	**Men**	**Women**
	(*n* = 3,200)	(*n* = 1,474)	(*n* = 1,726)
	Mean (SD)	Mean (SD)	Mean (SD)
Age (years)	39 (11)	40 (11)	39 (10)
Weight (kg)	71 (16)	81 (14)	63 (12)
Height (cm)	167 (9)	174 (7)	161 (7)
BMI (*kg*/*m* ^2^)	25 (5)	27 (4)	24 (5)
*BMI categories*			
Underweight (% of participants)	9.5	3.1	15.0
Normal weight (% of participants)	43.7	34.7	51.3
Overweight (% of participants)	32.3	43.6	22.8
Obese (% of participants)	14.5	18.7	10.9
BAI	29 (5)	27 (4)	30 (5)
% Fat CUN-BAE	29.8 (7.8)	25.4 (6.6)	33.5 (6.7)
Hip circumference (cm)	100 (9)	102 (8)	99 (10)
Waist circumference (cm)	87 (13)	94 (12)	81 (11)
% Fat BIO	28 (8)	24 (7)	32 (7)

Underweight (BMI < 18.5*kg*/*m* ^2^); Normal weight (BMI 18.5 ≤ 25*kg*/*m* ^2^); Overweight (BMI 25 ≤ 30*kg*/*m* ^2^); Obese (BMI ≥ 30*kg*/*m* ^2^).

Characteristics of participants in the study categorized by gender are also shown in Table 1. In the whole sample (S1 Dataset), BAI and BF showed a strong correlation (*ρ* = 0.74), being higher than the one between BMI and BF (*ρ* = 0.54); after categorization by gender the correlation between BAI and BF became slightly weaker (*ρ* = 0.71 for women; *ρ* = 0.68 for men), and the correlation between BMI and BF became stronger (*ρ* = 0.80 for women; *ρ* = 0.80 for men). In the sample of overweight and obese subjects (dataset B), BAI and BF showed a strong correlation (*ρ* = 0.77), higher than the one between BMI and BF (*ρ* = 0.51); after categorization by gender correlation between BAI and BF became weaker (*ρ* = 0.63 for women; *ρ* = 0.57 for men), and correlation between BMI and BF became stronger (*ρ* = 0.72 for women; *ρ* = 0.73 for men). Table 2 shows the multi regression models obtained.

**Table 2:** Multi regression models obtained to predict BF, and the different correlations between the model and BF obtained using BIA.

MODELS	Body Fat ∼	*R* ^2^	Error	*ρ*
Model 1a	−53.03 + (57.99 × *f*(*BMI*))	29%	6.88	0.54
Model 1b	−91.15 + (82.09 × *f*(*BAI*))	56%	5.44	0.75
Model 1c	−78.62 + (75.41 × *f*(*BMI*))	26%	6.46	0.51
Model 1d	−86.63 + (79.50 × *f*(*BAI*))	59%	4.80	0.77
Model 2a	−61.50 + (53.01 × *f*(*BMI*)) + (9.79 × *f*(*Age*))	31%	6.78	0.56
Model 2b	−98.70 + (78.72 × *f*(*BAI*)) + (7.89 × *f*(*Age*))	57%	5.36	0.75
Model 2c	−87.35 + (75.18 × *f*(*BMI*)) + (5.62 × *f*(*Age*))	27%	6.43	0.52
Model 2d	−95.83 + (79.42 × *f*(*BAI*)) + (5.77 × *f*(*Age*))	60%	4.76	0.78
Model 3a	−96.07 + (76.91 × *f*(*BMI*)) + (6.65 × *f*(*Age*)) + (11.40 × *Sex*)	75%	4.09	0.87
Model 3b	−87.68 + (67.20 × *f*(*BAI*)) + (10.06 × *f*(*Age*)) + (4.37 × *Sex*)	62%	5.01	0.79
Model3c	−90.99 + (74.25 × *f*(*BMI*)) + (6.05 × *f*(*Age*)) + (11.02 × *Sex*)	78%	3.54	0.88
Model3d	−61.17 + (54.15 × *f*(*BAI*)) + (6.16 × *f*(*Age*)) + (5.89 × *Sex*)	69%	4.22	0.83
Model 4a	785.58 − (563.95 × *f*(*Age*)) − (27.12 × *Sex*)−	75%	4.07	0.87
	(1199.65 × *f*(*BMI*)) +			
	(461.04 × *f*(*BMI*)^2^) +			
	(63.08 × *f*(*BMI*) × *Sex*) +			
	(822.11 × *f*(*BMI*) × *f*(*Age*))−			
	(25.31 × *f*(*BMI*)^2^ × *Sex*)−			
	(295.51 × *f*(*BMI*)^2^ × *f*(*Age*))			
Model 4c	−1854.17 − (1082.08 × *f*(*Age*)) − (141.86 × *Sex*) +	79%	3.48	0.89
	(2321.40 × *f*(*BMI*))−(710.71 × *f*(*BMI*)^2^) +			
	(217.23 × *f*(*BMI*) × *Sex*)−			
	(1373.39 × *f*(*BMI*) × *f*(*Age*))−			
	(76.99 × *f*(*BMI*)^2^ × *Sex*) +			
	(435.11 × *f*(*BMI*)^2^ × *f*(*Age*))			
Model 4’a	770.32−(565.46 × *f*(*Age*)) − (1178.25 × *f*(*BMI*)) +	75%	4.07	0.87
	453.57 × *f*(*BMI*)^2^ + (824.15 × *f*(*BMI*) × *f*(*Age*)) +			
	(24.95 × *f*(*BMI*) × *Sex*)−			
	(11.94*f*(*BMI*)2 × *Sex*)−			
	(296.20 × *f*(*BMI*)2 × *f*(*Age*))			
Model 4’c	−1887.67 − (1058.38 × *f*(*Age*)) +	79%	3.47	0.89
	(2365.27 × *f*(*BMI*))−			
	(724.96 × *f*(*BMI*)^2^) + (27.26 × *f*(*BMI*) × *Sex*)−			
	(1340.88 × *f*(*BMI*) × *f*(*Age*))−			
	(13.51 × *f*(*BMI*)^2^ × *Sex*) +			
	(423.95 × *f*(*BMI*)2 × *f*(*Age*))			
CUNBAE a	−44.988 + (0.503 × *Age*) + (10.689 × *Sex*) +			0.86
	(3.172 × *BMI*)−(0.026 × *BMI* ^2^) +			
	(0.181 × *BMI* × *Sex*)−(0.02 × *BMI* × *Age*)−			
	(0.005 × *BMI* ^2^ × *Sex*) + (0.00021 × *BMI* ^2^ × *Age*)			
CUNBAE c	−44.988 + (0.503 × *Age*) + (10.689 × *Sex*) +			0.89
	(3.172 × *BMI*)−(0.026 × *BMI* ^2^) +			
	(0.181 × *BMI* × *Sex*)−(0.02 × *BMI* × *Age*)−			
	(0.005 × *BMI* ^2^ × *Sex*) + (0.00021 × *BMI* ^2^ × *Age*)			

BIA: Bioelectrical Impedance Analysis.

Models a and b have been obtained with the whole dataset (S1 Dataset), and models c and d have been obtained with the whole dataset (S1 Dataset) constrained to overweight/obese subjects (datasetB). Models a and c include BMI as predictor variable, models b and d include BAI as predictor variable.

Using the general eqs (1) and (2) we first fit two regression models relating BF to i) BMI (model 1a) and ii) BAI (model 1b) using S1 Dataset. The same process was performed using dataset B (restricted to overweight/obese) obtaining models 1c and 1d for BMI and BAI respectively:

$$\begin{array}{ccc} BF & = & a_{0}+a_{1}f\left(BMI\right)+e \end{array}$$ (1)

$$\begin{array}{ccc} BF & = & b_{0}+b_{1}f\left(BAI\right)+e \end{array}$$ (2)

where *a*_0_ and *b*_0_ are intercept terms and a1 and b1 represent the change in body fat (BF) associated with a change of 1 unit in logarithm of body mass index (*f*(*BMI*)), and with a change of 1 unit in logarithm of body adiposity index (*f*(*BAI*)), respectively. Model 1*a* explains 29% of the variability in BF (model 1c explains 26%), whereas model 1*b* explains 56% (model 1*d* explains 59%), showing BAI as a better predictor of BF when it is considered as the only variable in the equation. To these models we add age (logarithmically transformed) variable as a new predictor obtaining:

$$\begin{array}{ccc} BF & = & a_{0}+a_{1}f\left(BMI\right)+a_{2}f\left(Age\right)+e \end{array}$$ (3)

$$\begin{array}{ccc} BF & = & b_{0}+b_{1}f\left(BAI\right)+b_{2}f\left(Age\right)+e \end{array}$$ (4)

Model 2*a* explains 31% of the variability in BD (model 2*c* explains 27%), whereas model 2*b* explains 57% (model 2*d* explains 60%), showing again BAI as a better predictor of body fat. The addition of the Sex variable (where male = 0 and female = 1 for Sex variable as it is in the CUN-BAE [eq 19]) to eqs (3) and (4), leading to eqs (5) and (6) respectively, produced different effects.

$$\begin{array}{ccc} BF & = & a_{0}+a_{1}f\left(BMI\right)+a_{2}f\left(Age\right)+a_{3}Sex+e \end{array}$$ (5)

$$\begin{array}{ccc} BF & = & b_{0}+b_{1}f\left(BAI\right)+b_{2}f\left(Age\right)+b_{3}Sex+e \end{array}$$ (6)

**Fig 1:**
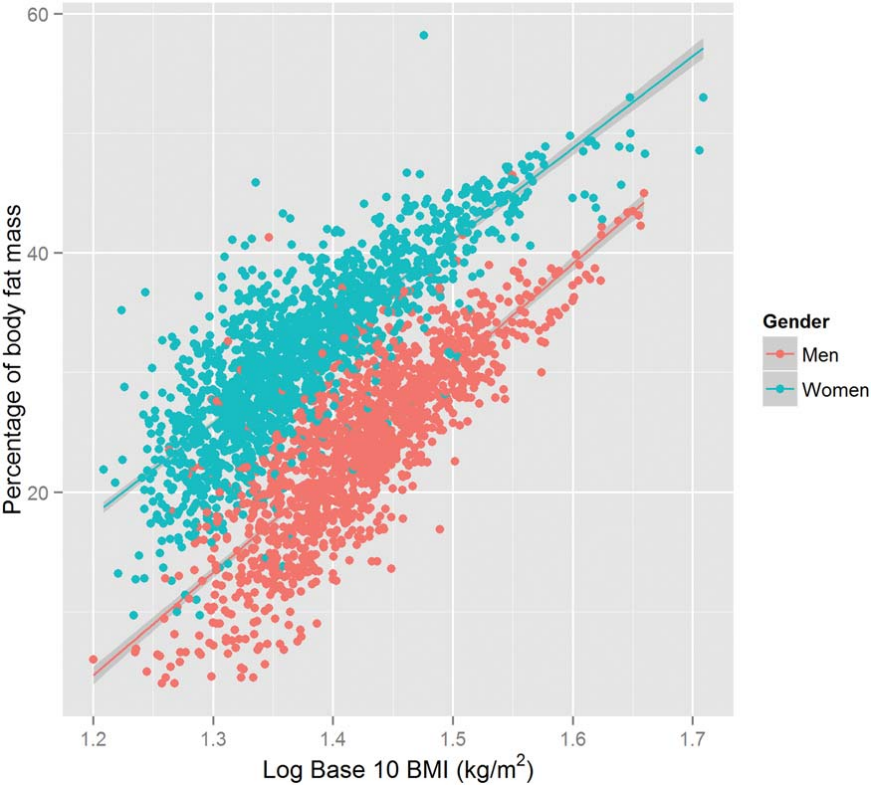
Regression from linear model 3a relating the percentage of body fat to Log 10 BMI (Body Mass Index)(*kg*/*m* ^2^). The regression model 3a adjusted for a 2-level factor variable for Sex, and age. The points are coloured by Sex groups.

The model obtained using eq (5) explains 75% of variability (model 3a, which is shown in Fig 1), and 78% of variability (model 3c), whereas model expressed in eq (6) explains 62% of variability (model 3b), and 69% of variability (model 3d).

**Fig 2:**
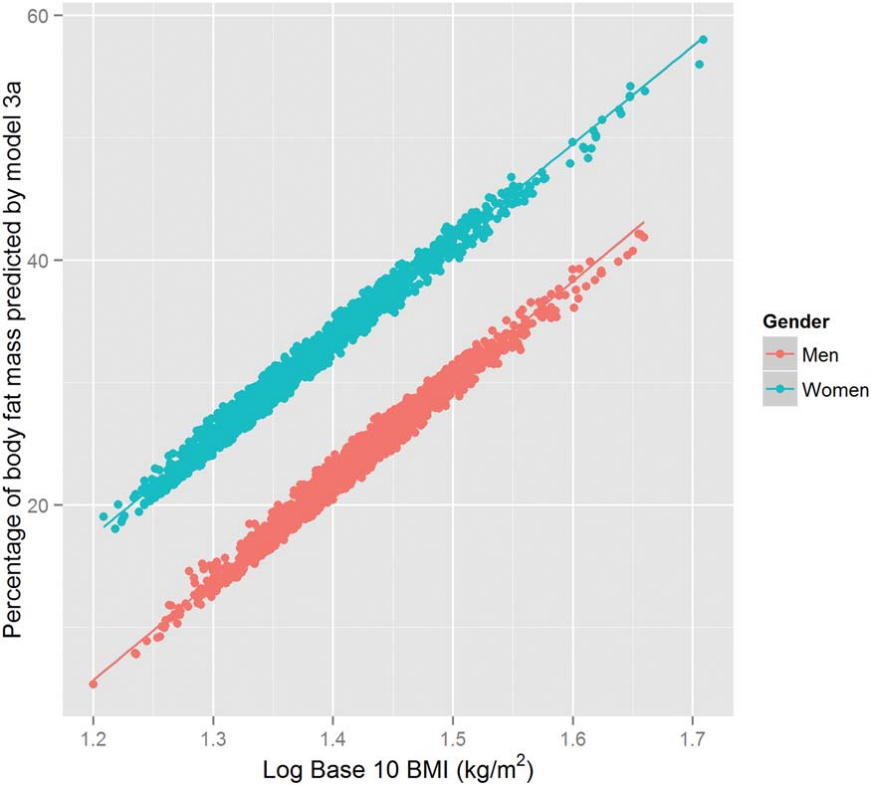
Predicted model from linear model 3a relating the predicted percentage of body fat to Log 10 BMI (Body Mass Index)(*kg*/*m* ^2^). The predicted model 3a is adjusted for a 2-level factor variable for Sex, and age. The points are coloured by Sex groups.

A predicted model from eq (5) and model 3a is represented in Fig 2.

**Fig 3:**
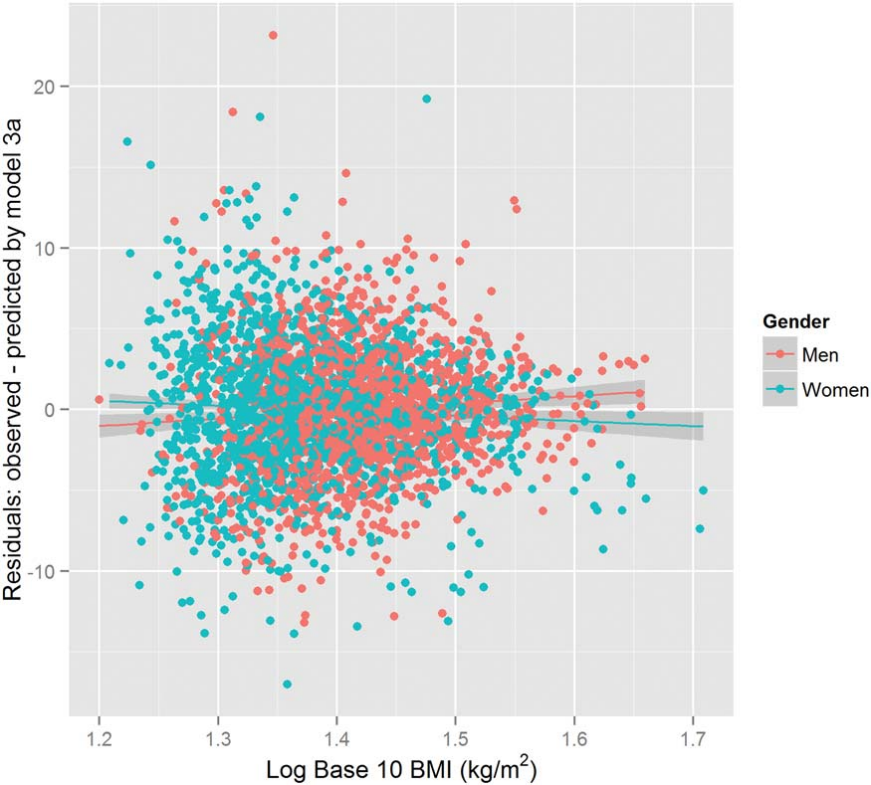
Residuals from linear model 3a relating observed—predicted percentage of body fat to Log 10 BMI (Body Mass Index)(*kg*/*m* ^2^). The residuals are adjusted for a 2-level factor variable for Sex, and age in model 3a. The residuals are coloured by Sex groups.

Regression model 3a appeared to remove most of the non-random patterns of variation in the residuals (see Fig 3).

The error term e represents all sources of unmeasured and unmodeled random variation in body fat (BF). Following with this study, we also consider a regression model relating BF as a target variable to BMI, sex, age and their interactions as the predictor variables (model 4a, and model 4c).

$$\begin{array}{ccc} BF & = & a_{0}+a_{1}f\left(BMI\right)+a_{2}f\left(Age\right)+a_{3}Sex+a_{4}f{\left(BMI\right)}^{2}+\\ & & a_{5}f\left(BMI\right)\times Sex+a_{6}f\left(BMI\right)\times f\left(Age\right)+\\ & & a_{7}f{\left(BMI\right)}^{2}\times Sex+a_{8}f{\left(BMI\right)}^{2}\times f\left(Age\right)+e \end{array}$$ (7)

Models derived from eq (7) explain 75% of variability (model 4a) for the whole group of participants, which is similar to the value obtained using model 3a, in spite of model 4a was more complex, and 79% of variability (model 4c) for the overweight and obese subjects, showing a small improvement with respect to model 3c. Looking at the significance of some of the coefficients, we may question the inclusion of some of them in models 4a and 4c. To simplify the regression model we apply backward elimination using the step() function in R [20], this function uses the Akaike Information Criterion (AIC) to perform model search. By backward elimination method the final regression model was:

$$\begin{array}{ccc} BF & = & a_{0}+a_{1}f\left(BMI\right)+a_{2}f\left(Age\right)+a_{3}f{\left(BMI\right)}^{2}\\ & & +a_{4}f\left(BMI\right)\times Sex+a_{5}f\left(BMI\right)\times f\left(Age\right)+\\ & & a_{6}f{\left(BMI\right)}^{2}\times Sex+a_{7}f{\left(BMI\right)}^{2}\times f\left(Age\right)+e \end{array}$$ (8)

Model expressed in eq (8) explains 75% of variability (model 4’a) similar to model 3a and 4a; and 79% of variability (model 4’c) when only overweight and obese subjects (dataset B) were considered, showing a bit improvement with respect to model 3c.

### Validation

The correlation between Model 3a and BF obtained using BIA in the validation sample (S2 Dataset) was *ρ* = 0.77 and, between CUN-BAE equation and BF we obtained the same correlation (*ρ* = 0.77). Overweight and obese subjects were also selected from this sample, obtaining a new dataset of 1,379 subjects (373 women, 1,006 men), with a mean age of 46.5 years (SD 7.8). In the sample of overweight and obese subjects the correlation between BF and Model 3a was *ρ* = 0.76 and, between BF and CUN-BAE equation *ρ* = 0.77 slightly higher than Model 3a. After that, the whole dataset(2,153 observations) was stratified by sex, dividing it in two datasets (men n = 1,400 and women n = 753). In the men dataset correlation between BF and either Model 3a or CUN-BAE equation was the same (*ρ* = 0.74). In the women dataset correlation between BF and either Model 3a or CUN-BAE equation was also the same (*ρ* = 0.81). We proceed in a similar way considering the dataset of overweight/obese subjects (n = 1,379), the dataset was divided in two, one composed by men (n = 1,006), and another composed by women (n = 373). In the men one, correlation between BF and either Model 3a or CUN-BAE equation was for both equations *ρ* = 0.67. In the women dataset the correlation between BF and Model 3a was *ρ* = 0.68, and correlation between BF and CUN-BAE equation was *ρ* = 0.69.

## Discussion

The main finding of the present study was that using simpler equations than previously suggested, an equally approximate estimation of BF can be obtained. Furthermore, when it is considered as the only variable in the equation, the BAI has been revealed as a better BF predictor than the BMI. Several multiple regression models to predict BF are presented using either BMI or BAI as indicators of BF. Both indicators have been shown to be useful in epidemiologic studies for estimating BF. However, both BMI and BAI present important limitations when a proper BF estimation is required and several equations for predicting BF have been suggested using mainly BMI as the primary variable. In these equations variables such as age and sex are used to increase the accuracy. However, it has been reported that most of these equations has been derived from small samples or from imprecise methods of body fat measurement [9–12]. In order to prevent these limitations, the CUN-BAE equation, which uses BMI as the main variable, was developed as an attempt to increase the accuracy in BF estimation [12]. However, this equation was derived from a sample of mainly overweight and obese subjects, which could lead to inaccuracies when applied to a sample with different characteristics. In the present study several prediction equations with increasing complexity were derived from both the whole sample of participants and the sample obtained selecting the overweight and obese participants to allow a proper comparison with the CUN-BAE equation. From the first analysed regression models, expressed in eq (1) and eq (2) using, respectively, only BMI or BAI as variables, the BAI was revealed as a better variable for the prediction of BF as the model derived from eq (2) explains more percentage of variance. This result could be in agreement with the suggestion that BMI does not consider the sexual dimorphism characteristics of body adiposity. In fact, this is only one of the limitations of BMI. When BMI is used, it should be considered that it does not take into account ethnicity or age of individuals, it is not applicable to people with high fitness and also takes into account bone mass, lateral and anteroposterior size, the relative proportion trunk/limbs, etc. [5, 6]. However, it is considered that hip circumference, which is included in the BAI calculation, captures male-female differences in adiposity better than the BMI [21]. Thus, the inclusion of the BAI in this simple equation supposes an important conceptual advantage over the inclusion of the BMI because differences between men and women regarding adiposity are reflected more properly using the BAI than the BMI. With the aim to improve the adjustment of the prediction equations the variable Age was included, giving models 2a, 2b, 2c and 2d. But the addition of this variable did not suppose a significant change in the accuracy of the equations.

The lack of changes in the accuracy of the equation when the age was included is a controversial result because others have suggested that the inclusion of age is essential for improving the accuracy of equations in spite of the contribution of this variable was not determined [19]. In fact, it has been reported that the relation between BMI and BF is dependent on age [8] and older adults have on average more body adiposity than younger adults at any given BMI [5]. Furthermore, it has been reported that the aging process brings about many changes in body composition, such as an increase in adiposity and a decrease in water content, often without concomitant changes in BMI [22]; in fact, as individual age, fat mass increase and lean tissue or muscle mass tends to decrease and lipids infiltrates other non-adipose tissue stores, such as liver. These changes have repercussions not only on health but also on the methods to assess body composition. The extent of these changes varies depending on age, gender and ethnicity, and may be mediated by lifestyle factors [23]. The lack of effect when age is taken into account could be related to the age of participants in the present study because participants older than 65 years were not considered, meanwhile other studies, such the one of the CUNBAE considered participants as old as 80 years [19]. The fact that a positive correlation was found between BMI and age, at least in women, could also contribute to the lack of effect when age is considered [5]. However, the addition of the Sex variable, giving models 3a, 3b, 3c and 3d, produced a slight improvement in adjustment of BAI based models but a great improvement in the BMI based models, becoming BMI a better main variable than BAI when the Sex is considered in the equation. In fact, models using BMI report 75% of variance (model 3a, obtained with the whole sample: S1 Dataset) and 78% of variance (model 3c, using the sample of overweight and obese subjects: dataset B). This last result could be in agreement with the previous one showing the BAI as a better indicator of BF when it is considered as the only variable in the equation because the BAI itself considers, as it has been indicated above, the sexual dimorphism in adiposity. On the other hand, when the equation accounts for the Sex variable in predicting BF, BMI becomes a better predictor of BF, which is in agreement with previous results from our group showing that, when categorized per sex, correlation coefficients between BMI and BF for both men and women were higher than the ones between BAI and BF [7]. Therefore, similar to other studies our results show that sex is a necessary variable to determine BF using BMI. CUN-BAE equation explained the 79% of variability in the original study [19]. This equation considers the Sex as a variable which, in agreement with previous comments, certainly will contribute to increase the variance explained. However, when the CUN BAE equation was tested in the whole sample of the present work a correlation of *ρ* = 0.86 was obtained similar to the one obtained with the simple model 3a (*ρ* = 0.87). When the sample of overweight and obese subjects (dataset B) is considered, the simple model 3c shows a *ρ* = 0.88, very similar to the correlation obtained by the CUN-BAE equation in the sample of overweight and obese subjects (dataset B) *ρ* = 0.89. These last results suggest that in spite of the CUN-BAE equation was derived from a sample of mainly overweight and obese subjects, this does not suppose a better behavior when it is applied to another sample of similar characteristics. As occurs with the original CUNBAE equation and others, the main limitation of the present study could be that only Caucasian participants were considered. Therefore, the applicability of the equations obtained should be tested in other populations. In fact, the equations reported in the literature have been applied to an only ethnic group because of the differences in adiposity found among different groups. Thus, because the approximations used in the equations try to adjust per different characteristics of the population involved, it is unclear whether an only equation will give better, or similar, results than any of the experimental measures widely shown in the literature. However, the main strength of these equations is that an estimation of the body composition could be obtained within a population using simple data such as weight, height and gender.

A limitation of the present study was that reference values were measured using BIA. In spite of it is well-accepted, BIA measurements present some inaccuracies because they are affected by body position, hydration status, consumption of food and beverages, ambient air and skin temperature, recent physical activity, and conductance of the examining table. This methodology presents also intra-individual differences, particularly in relation to the proportions of water and mineral in the fat-free compartment. This variability contributes to the absolute error of the method [24].

## Conclusions

From the present study we conclude that there are simpler models than CUN-BAE equation that fits BF as well as CUN-BAE does. Therefore, it could be considered that CUN-BAE overfits and it violates the principle of parsimony because the need for this more complex equation is not justified. In Clinical Practice, simple models are easier to understand, implement, and use so, it seems that the use of complex equations such as the CUN-BAE equation is not justified. We also conclude that when using a simple linear regression model, the BAI, as the only variable, predicts BF better than BMI. However, when the Sex variable is introduced in the model, BMI becomes the indicator of choice to predict BF.

## Supporting Information

S1 Dataset Includes all participants in the study (n = 3,200). (TXT)

S2 Dataset Includes all participants in the study for validation (n = 2,153). (TXT)
